# Evaluation of Marker Compounds and Biological Activity of In Vitro Regenerated and Commercial *Rehmannia glutinosa* (Gaertn.) DC. Roots Subjected to Steam Processing

**DOI:** 10.1155/2022/1506703

**Published:** 2022-12-12

**Authors:** Endang Rahmat, Yuseong Chung, Hyeon Hwa Nam, A. Yeong Lee, Jun Hong Park, Youngmin Kang

**Affiliations:** ^1^University of Science & Technology (UST), KIOM Campus, Korean Convergence Medicine Major, Daejeon 34054, Republic of Korea; ^2^Herbal Medicine Resources Research Center, Korea Institute of Oriental Medicine, 111 Geonjae-ro, Naju-si, Jeollanam-do 58245, Republic of Korea

## Abstract

*Rehmannia glutinosa* (Gaertn.) DC., belonging to the family Scrophulariaceae, has been known since immemorial times as a prominent oriental drug in East Asia that can treat various ailments, such as kidney disorders, anemia, and diabetes. In order to be applied for medical purposes, *R. glutinosa* is commonly processed using steam to increase its efficacy and biological activity. The increasing demand for *R. glutinosa* in the traditional medicine industry encouraged many researchers to develop a fast, efficient, and high-quality production system using biotechnological approaches. This study aimed to compare the chemical and biological activities of in vitro regenerated *R. glutinosa* (PKR) and commercial *R. glutinosa* (PCR) samples subjected to steam processing. We assessed the effects of steam processing and the differences in *R. glutinosa* material on 5-Hydroxymethyl-2-furaldehyde (5-HMF) content, total flavonoid and phenolic content, antioxidant activity, nitric oxide (NO) levels, and anti-inflammatory activity. PKR samples showed a significantly higher content of 5-HMF (0.15%) as compared to PCR samples (0.05%). Compared to unprocessed *R. glutinosa* (UPR) and PCR samples, PKR again showed the highest total phenolic and flavonoid content of 41.578 mg GAE/g and 17.208 mg RUE/g, respectively. Meanwhile, both processed *R. glutinosa* samples (PKR and PCR) showed a significantly higher DPPH antioxidant activity ((67.095 + 1.005)% and (61.579 + 0.907)%, respectively) than unprocessed *R. glutinosa* ((31.452 + 1.371)%). In addition, both PKR and PCR samples showed good anti-inflammatory activity by showing similar effects such as the inhibition of NO production and the suppression of *inducible nitric oxide synthase* (iNOS). Based on these results, PKR fulfilled the Chinese pharmacopeia standards, in terms of the amount of the marker compounds and showed a high level of bioactivity. Therefore, these findings are expected to be useful in verifying the efficacy of herbal medicines and the availability of suitable materials for medicinal use.

## 1. Introduction

*Rehmannia glutinosa* (Gaertn.) DC. (Orobanchaceae) is a highly important medicinal plant belonging to the family Scrophulariaceae. It is considered a “top grade” herb in traditional medicine in eastern countries such as Korea and China. *R. glutinosa* is traditionally believed to nourish *Yin* and tonify the kidneys, including the regulation of water metabolism, reproduction and development, dominating growth, storing essence, and regulation of the neuroendocrine system using the traditional Chinese medicine (TCM) theory, showing that *R. glutinosa* contains various types of active compounds and exhibits several pharmacological benefits [1]. There are three types of *R. glutinosa* samples used based on their processing methods as follows: fresh *R. glutinosa* roots (Xian Dihuang), dried *R. glutinosa* roots (Sheng Dihuang), and processed *R. glutinosa* roots (Shu Dihuang). Several essential phytochemicals have been isolated and identified pharmacologically from unprocessed (raw) and processed *R. glutinosa* including catalpol, verbascoside, and 5-Hydroxymethyl-2-furaldehyde (5-HMF).

Pharmacologically, unprocessed and processed *R. glutinosa* have shown to possess several biological activities, such as inhibition of tumoral processes (antitumor activity) through their action on the enzyme topoisomerase I [2] and are toxic to the lung cancer cell lines (cytotoxicity) [3]. Specifically, catalpol shows extensive protection for neuronal ischemia [4], elevates brain angiogenesis [5], and possesses hypoglycemic and diuretic abilities [1]. On the other hand, harpagide shows leishmanicidal activity [6] and is capable of downregulating the production of enzymes, such as lipopolysaccharide-induced nitric oxide synthase and cyclooxygenase-2 (COX-2) through suppression of nuclear factor *κ*B [7]. *R. glutinosa* roots also accumulate phenylethanoid glycosides, including verbascoside and isoverbascoside, which are proven to show various medicinal values, including cytotoxic and apoptotic activity against breast cancer cell lines [8].

The constituents extracted from the root and above-ground biomass of medicinal herbs contain secondary metabolites, also referred to as phytochemicals, encompass a large variety of natural products including alkaloids, glycosides, phenols, flavonoids, terpenoids, saponins, steroids, tannins, quinones, and coumarins [9]. Herbs can be successfully engineered as biofactories for synthesizing biomolecules with pharmaceutical and industrial interest, while these achievements require a thorough understanding of biochemical knowledge to amend large-scale production [10]. The increasing demand for *R. glutinosa* herb in traditional medicine has encouraged many researchers to develop a fast, efficient, and high-quality production system. One of them is the application of biotechnological propagation to obtain an optimum growth system to produce large-scale*R. glutinosa*. Previously, our research group developed an optimum in vitro propagation protocol for the high-quality production of *R. glutinosa* seedlings and rootstocks [11] which has been patented under patent number 10-1881305. Our protocol has also been evaluated in a large field farming system and was able to produce greater rhizome biomass than the commercially available *R. glutinosa* seedlings [12]. To provide high-quality processed *R. glutinosa* for commercialization purposes in the traditional medication system, we further evaluated our processed *R. glutinosa* products by comparing the roots of *R. glutinosa* obtained from in vitro and commercial plants. Here, we examined some pivotal parameters for herbal drug development, such as marker compound, antioxidant, and anti-inflammatory properties.

## 2. Materials and Methods

### 2.1. Collection of *R. glutinosa* Root Samples

**Figure 1:**
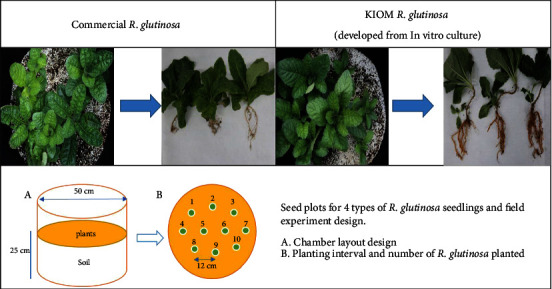
Cultivation process of commercial *R. glutinosa* and in vitro regenerated *R. glutinosa* indoor KIOM production and propagation laboratory in Naju, Jeollanam-do, South Korea.

We compared two different *R. glutinosa* root samples (Figure 1). The first one was the in vitro regenerated *R. glutinosa* roots, a patented sample based on in vitro propagation protocol developed by the Korea Institute of Oriental Medicine (KIOM) [11]. The optimized in vitro propagation protocol consists of WPM + IAA 1.0 mg/L + IBA 0.5 mg/L combination. The sample was harvested from the indoor KIOM production and propagation laboratory in Naju, Jeollanam-do, South Korea. Secondly, commercial *R. glutinosa* roots, commercially available as *R. glutinosa* sample, was harvested from an indoor KIOM production and propagation laboratory in Naju, Jeollanam-do, South Korea. All samples were collected 6 months after planting in the commercially available soil.

### 2.2. Processing of *R. glutinosa* Roots

**Figure 2:**
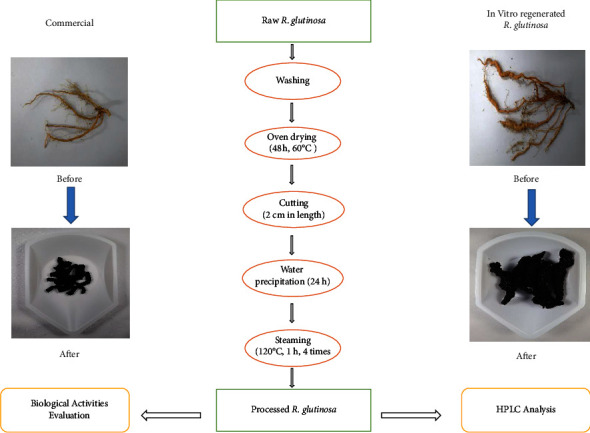
The overall methodology for processing of *R. glutinosa* roots by steaming treatment.

Raw *R. glutinosa* root samples (in vitro regenerated and commercial) were washed using distilled water and dried in an oven at 60°C for 48 h. All the dried samples were then cut into a length of 2 cm and soaked in water to separate the bad samples. All samples were steamed (KSP-240 L, Kyungseo E&P, South Korea) for 1 h at 120°C four times (Figure 2). This optimized steam processing method was developed and patented by Korea Institute of Oriental Medicine (KIOM). After processing, three different types of samples were obtained for further analysis, namely, unprocessed *R. glutinosa* (UPR), processed commercial *R. glutinosa* (PCR), and processed in vitro *R. glutinosa* (PKR).

### 2.3. Samples Extraction

All samples (UPR, PCR, and PKR) were ground into powder using a steel pulverizing machine (250G New Type Pulverizing Machine, Model RT-N04-2V, Taiwan) at 25,000 rpm. The maceration method was applied to extract 2 g of fine powder from each sample in 35 mL of 70% ethanol (JT Baker Inc., Phillipsburg, NJ, USA), followed by sonication at 40°C for 1 h. All extracts were filteredusing a 0.45 *μ*m syringe filter (PALL Corporation, Ann Arbor, MI, USA) and concentrated in a reduced pressure rotary evaporato(EYELA N-1200B, Tokyo Rikakikai Co. Ltd., Japan) at 40°C. Each concentrated extract was then vacuum-dried.

### 2.4. DPPH Antioxidant Measurement

Approximately, 50 mg of each concentrated extract sample was dissolved in 5 mL 70% ethanol to obtain a stock solution (10000 *μ*g/mL). Different concentrations (125, 250, 500, and 1000 *μ*g/mL) of each sample were prepared by diluting the stock solution for antioxidant testing.

The antioxidant activity of all samples (UPR, PCR, PKR) was established using 1,1-diphenyl-2-picrylhydrazyl (DPPH, Sigma-Aldrich (St. Louis, MO, USA)), adapted from Okello et al. [13]. About 0.1 mL of each sample (at different concentrations) was added to 0.1 mL of 200 *μ*M DPPH solution (Sigma-Aldrich, St. Louis, MO, USA), wherein gallic acid was used as the positive control. The mixture was then incubated for 30 min at 37°C. Absorbance analysis was performed at a wavelength of 517 nm using a spectrophotometer (Spectramax i3x (Molecular Devices, Wokingham, UK)). The radical scavenging ability of these samples was calculated as follows:

(1) $$\begin{array}{c} DPPH\;activity\left(\%\right)=\frac{\left[control\;absorbance\;–\;sample\;absorbance\right]}{control\;absorbance}\times 100\text{.} \end{array}$$

Moreover, a simple regression analysis was performed to calculate the value of IC_50_.

### 2.5. Total Flavonoid

The total flavonoid content was obtained by modifying the methodology of Okello et al. [13]. 90% diethyl glycol (0.8 mL) and 1 N sodium hydroxide (10 *μ*L) were mixed with 0.1 mL of each sample extract (1000 ppm concentration) in a 1.5 mL microcentrifuge tube. A vortex was then used to homogenize the solution, and a water bath system was used for incubation (60 min, 37°C). Sample absorbance was measured using a spectrophotometer (Spectramax i3x (Molecular Devices, Wokingham, UK)) in triplicates at 420 nm wavelength. A calibration curve between the standard and rutin was constructed to obtain the total flavonoid content as mg rutin equivalent (Rue)/g.

### 2.6. Total Phenolic

Total phenolic content was measured using the modified methodology of Derakhshan et al. [14]. A Folin−Ciocalteu's reagent was added to a 0.5 mL sample solution (0.3 mg/mL) in a 1.5 mL tube. A 10% Na_2_CO_3_ (0.5 mL) solution was added to the mixture, followed by incubation for 60 min at 25°C in the dark. Absorbance was measured at 725 nm using a spectrophotometer (Spectramax i3x (Molecular Devices, Wokingham, UK)). The TPC was determined by interpolation of the standard curve using gallic acid with other samples. The total phenolic content was presented as mg gallic acid equivalent (GAE) g^−1^.

### 2.7. HPLC Analysis

All concentrated sample extracts (100 mg each) were dissolved in 80% HPLC-grade methanol (1 mL) and sonicated for approximately 1 h. Samples were filtered using a 0.22 *μ*M syringe filter (PALL Corporation, Ann Arbor, MI, USA) and placed in an HPLC tube. HPLC analysis was used to determine the 5-HMF and verbascoside content. Approximately, 1 mg of each 5-HMF and verbascoside was dissolved in 80% methanol to form a 10,000 ppm standard solution. HPLC analysis was performed using an HPLC machine (Waters Corporation, Milford, MA, USA) with a 2695 separation module and 2996 photodiode array detector. A Waters Capcell Pak UG120 C18 analytical column (250 × 4.6 mm, 5 *μ*m; Shiseido, Japan) was used for separation at 30°C. The mobile phase consisted of HPLC grade water with 0.1% formic acid (solvent A) and HPLC grade acetonitrile with 0.1% formic acid (solvent B) was used. The gradient program was set as follows: 0–15 min, 95% A; 15–25 min, 85% A; 25–45 min, 70% A; 45–60 min, 95% A. For bride sample analysis, the column was equilibrated using 99% B for 10 min. The flow rate was set to 0.8 ml/min with an injection amount of samples and standards set to 10 *μ*L, and the analytical wavelength set to 320 nm (verbascoside) and 280 nm (5-HMF). The detection of 5-HMF and verbascoside was performed by comparing their UV spectra, peak retention time, and atom masses for reference. The concentration of each compound in the samples was expressed as metabolites per gram of processed dried roots (mg/g DW).

### 2.8. Preparation of RAW 264.7 Cells and Cell Viability Assay

Culture of RAW 264.7 macrophage cell lines were done in Dulbecco's Modified Eagle Medium (DMEM) (Gibco, Invitrogen) supplemented with 1% (v/v) streptomycin/penicillin and 10% fetal bovine serum (FBS) and then incubated at 37°C in an atmosphere containing 5% CO_2_. The cell lines were then treated with the sample extracts at various concentrations (0, 100, and 200 *μ*g/mL) and further cultured with and without LPS (1 *μ*g/mL) for 24 h at 37°C. A cell counting kit (CCK-8, Dojindo, Kumamoto, Japan) assay was used to determine cell line proliferation on the substrates. All samples were seeded at a density of 2 × 10^5^ cells/mL per well in the 96-well plate. The absorbance was measured at 450 nm using ELISA.

### 2.9. Nitric Oxide (NO) Level Measurement

NO levels were determined following the modified methodology given by Choi et al. (2019). RAW 264.7 cells (2 × 10^5^ cells/mL) were pretreated with *R. glutinosa* extracts (0 and 200 *μ*g/mL) for 1 h, followed by lipopolysaccharide (1 *μ*g/mL) treatment for 24 h. After 24 h, the supernatant was collected to determine the NO levels. The Griess reaction method was used to measure nitrite accumulation as an indicator of NO value in the LPS-induced RAW 264.7 macrophage cell lines. Absorbance was measured using a spectrophotometer (Spectramax i3x (Molecular Devices, Wokingham, UK)) in triplicates at 540 nm.

### 2.10. Western Blot Analysis

Pretreatment of RAW 264.7 cell lines were pretreated with 200 *μ*g/mL sample extracts for 1 h followed by LPS stimulation (1 *μ*g/mL) for 24 h. All samples were seeded in a 6-well plate (1 × 106 cells/well). Treated cells were lysed using cold phosphate-buffered saline (PBS) and a lysis buffer containing protease inhibitors. The cell lysed were collected and centrifuged, and the protein concentration of the lysate was determined using a BCA™ protein assay kit. Equal amounts of protein were loaded onto an SDS-PAGE gel. Proteins were then transferred to a polyvinylidene fluoride membrane and incubated overnight at 4°C with specific primary antibodies at a dilution of 1/2000 in 5% w/v skimmed milk in tris buffered saline with tween 20 (TBST) followed by 1 h incubation with the HRP-conjugated secondary antibodies (1/2500 dilution in 1X TBST) at room temperature.

### 2.11. Statistical Analysis

One-way analysis of variance (ANOVA) was applied to all experimental data using Tukey's post hoc test using Prism (Graph Pad software, v5.03). Differences between compared means were considered statistically significant at *p* ≤ 0.05.

## 3. Results

### 3.1. Marker Compound Composition

**Figure 3:**
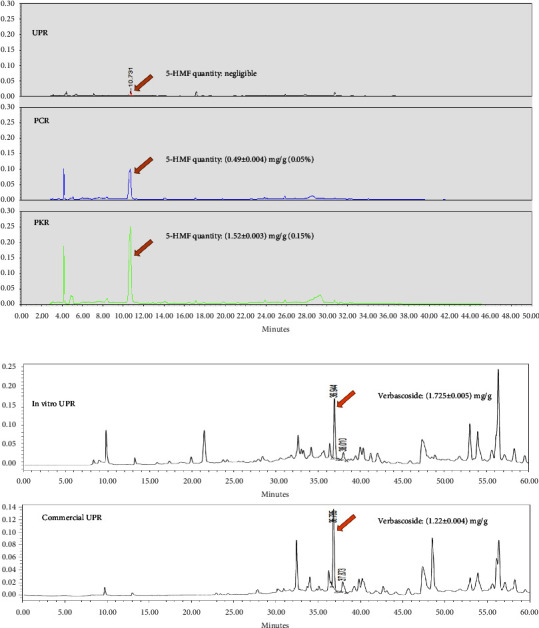
HPLC chromatogram and quantitative amount of marker compounds in all *R. glutinosa* roots sample extracts. (a) 5-HMF quantity in all *R. glutinosa* roots samples (b) Verbascoside content of in vitro UPR and commercial UPR samples.  ^*∗*^UPR, unprocessed *R. glutinosa*; PCR, processed commercial *R. glutinosa*; PKR, processed in vitro regenerated *R. glutinosa*. Values are presented as means ± standard deviation. Same letters are not significantly different by Tukey's test and *p*=0.05.

According to Chinese pharmacopoeia, the quality of processed *R. glutinosa* is determined by its 5-HMF content. The 5-HMF content of all *R. glutinosa* samples in this study was determined by comparing the HPLC retention time, UV absorption, and mass spectra with the standard (Figure 3(a)). As shown in Figure 3(a), 5-HMF was detected in PCR and PKR samples and was present in a very small amount (negligible) in the UPR sample. Further quantitative analysis of 5-HMF was performed at a wavelength of 280 nm. The highest 5-HMF content was observed in PKR (1.518 ± 0.004 mg/g). The results were significantly higher (*p*  <  0.05) than the rest of the *R. glutinosa* samples (Figure 3(a)). Furthermore, we assessed the content of verbascoside compound in unprocessed *R. glutinosa* roots (both in vitro and commercial samples) to evaluate the raw material quality. The result showed that in vitro UPR sample had slightly higher verbascoside content than commercial UPR sample (Figure 3(b)). This indicating higher quality of our in vitro *R. glutinosa* roots sample compared with commercially available *R. glutinosa* roots. Hence, this result becomes the basis foundation for development of processed *R. glutinosa* roots using in vitro-derived samples.

### 3.2. Antioxidant Activity

**Figure 4:**
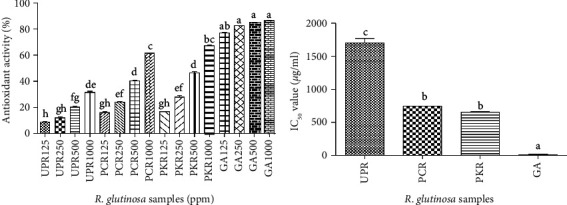
DPPH antioxidant activity of all type R. *glutinosa* sample extracts and gallic acid. (a) Percentage of antioxidant activity of all R. *glutinosa* extracts. (b) IC50 value of all R. *glutinosa* extracts.  ^*∗*^UPR, unprocessed R. *glutinosa*; PCR, processed commercial R. *glutinosa*; PKR, Processed in vitro regenerated R. *glutinosa*. Values are presented as means ± standard deviation. Same letters are not significantly different by Tukey's test and *p*=0.05.

The DPPH antioxidant activity of *R. glutinosa* samples was evaluated at different concentrations. The results showed that the antioxidant activity of all *R. glutinosa* samples increased as the concentration of the sample increased (Figure 4). Overall, the highest antioxidant activity was obtained in PKR (67.095%) and PCR (61.579%) samples at a concentration of 1000 mg/mL with the values of these two samples which were not significantly different (Figure 4(a)). Moreover, compared to the positive control (gallic acid, IC_50_ = 16.65 *μ*g/mL), the IC_50_ values of all these samples showed a lower DPPH radical scavenging effects (Figure 4(b)). Among all these samples, the PKR sample showed the lowest IC_50_ value (654.516 *μ*g/mL), although it was not significantly different from the PCR sample (749.646 *μ*g/mL), validating its high DPPH antioxidant activity.

### 3.3. Total Phenolic and Total Flavonoid Content

**Figure 5:**
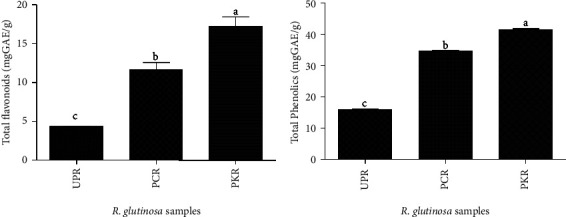
Total flavonoid and phenolic content in all *R. glutinosa* samples. (a) Total flavonoid content (b) Total phenolic content.  ^*∗*^UPR, unprocessed *R. glutinosa*; PCR, processed commercial *R. glutinosa*; PKR, processed in vitro regenerated *R. glutinosa*. Values are presented as means ± standard deviation. Same letters are not significantly different by Tukey's test and *p*=0.05.

The total phenolic and flavonoid concentration varies dramatically in all *R. glutinosa* samples (Figure 5(a)). The total phenolic compound ranged from 15.880 to 41.578 mg GAE/g, whereas the total flavonoid content ranged from 4.233 to 17.208 mg RUE/g (Figure 5(b)). PKR samples showed the highest total phenolic and flavonoid content of 41.578 mg GAE/g and 17.208 mg RUE/g, respectively. These values were significantly higher than the values obtained by PCR and UPR samples.

### 3.4. Effect of Samples Extracts on Cell Viability

**Figure 6:**
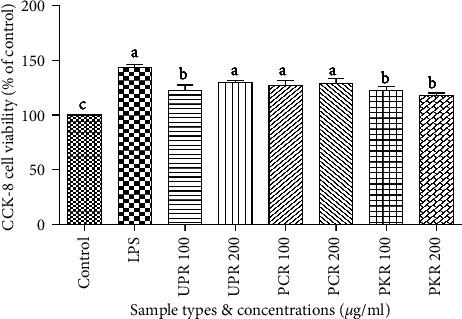
Effects of various *R. glutinosa* extracts on cell viability in RAW 264.7 macrophages cells. Cells were treated with concentrations (0, 100, and 200 *μ*g/ml) of various *R. glutinosa* extracts for 24 h. Cell viability was measured using the CCK-8 assay.  ^*∗*^UPR, unprocessed *R. glutinosa*; PCR, processed commercial *R. glutinosa*; PKR, processed in vitro regenerated *R. glutinosa*. Values are presented as means ± standard deviation. Same letters are not significantly different by Tukey's test and *p*=0.05.

The data showed that treatment with concentrations of 100 and 200 *μ*g/mL of all UPR, PCR, and PKR extracts resulted in the viability of cell lines compared with the control. There were no significant differences in cell viability among these samples (Figure 6).

### 3.5. Effects of Samples Extracts on NO Production Level

**Figure 7:**
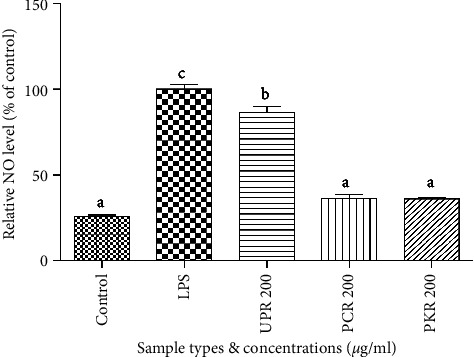
Inhibitory effects of all *R. glutinosa* samples extracts on NO production in LPS-induced RAW 264.7 cells at concentration of 200 *μ*g/ml.  ^*∗*^UPR, unprocessed *R. glutinosa*; PCR, processed commercial *R. glutinosa*; PKR, processed in vitro regenerated *R. glutinosa*. Values are presented as means ± standard deviation. Same letters are not significantly different by Tukey's test and *p*=0.05.

UPR, PCR, and PKR samples inhibited NO production in LPS-activated RAW 264.7 macrophage cell lines. Cells treated with processed *R. glutinosa* extracts (PCR and PKR) showed a stronger reduction in NO levels than the samples treated with unprocessed *R. glutinosa* extract (UPR) (Figure 7). However, there were no significant differences in NO levels after treatment with PCR and PKR (Figure 5).

### 3.6. Effects of Sample Extracts on the Expression of iNOS

**Figure 8:**
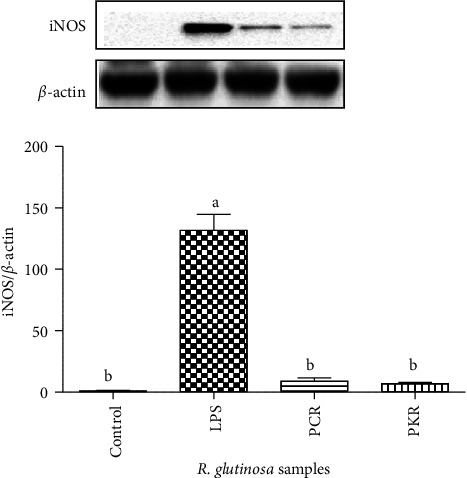
Inhibitory effect of R. *glutinosa* samples extracts on LPS-stimulated upregulation of iNOS expression in RAW 264.7 macrophages.  ^*∗*^UPR, unprocessed *R. glutinosa*; PCR, processed commercial *R. glutinosa*; PKR, processed in vitro regenerated *R. glutinosa*. Values are presented as means ± standard deviation. Same letters are not significantly different by Tukey's test and *p*=0.05. Control images were re-used for illustrative purposes.

iNOS expression was strongly inhibited by processed *R. glutinosa* samples (PKR and PCR) (Figure 8). However, there were no significant differences in iNOS inhibition levels between PKR and PCR assay. These results are in line with the inhibition of NO levels, which strongly suggest that the reduction in NO production is caused due to the suppression of iNOS expression.

## 4. Discussion

**Figure 9:**
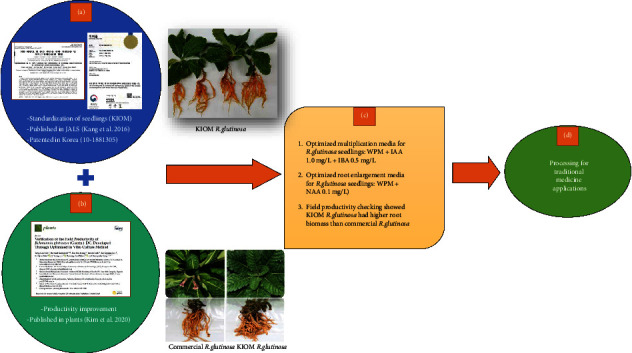
Summary of biotechnology-based optimization study to produce high-quality root material of *R. glutinosa* conducted in Korea Institute of Oriental Medicine (KIOM). (a) Standardization study of *R. glutinosa* seedlings using in vitro culture method. (b) Productivity validation study of *R. glutinosa* in large field, (c) summary of previous study results, (d) processing study as presented in this paper.

*R. glutinosa* roots have been used for centuries in China, Korea, and Japan as one of the top herbal medicines. It has several therapeutic benefits for a wide range of medical conditions. Commonly, there are three types of *R. glutinosa* roots in oriental medicine, namely, fresh *R. glutinosa* roots, dried *R. glutinosa* roots, and processed *R. glutinosa* roots by steam processing [15–17] and the addition of supplements. All these types of roots are used in different therapeutic applications based on traditional medicine theory and practices. Previously, our team conducted some optimization studies to produce good quality *R. glutinosa* roots using biotechnological approaches (Figure 9). The processing of *R. glutinosa* roots is aimed to reduce the toxicity and side effects to maximize the biological activities, change chemical properties or functions, preserve the active compounds, or maintain the purity from contaminants, such as microbes and other pathogens [18]. Although processed *R. glutinosa* roots have been studied extensively, there have been no studies using the roots developed biotechnologically in vitro which is the basic idea of this study. A previous study showed that *R. glutinosa* roots developed in vitro are safer than commercial ones since they are not contaminated by pathogens and contain higher active compounds [19].

Understanding the metabolic changes during processing is of great importance for the quality control of herbal drugs. Some previous studies have reported the change in the composition of active compounds in *R. glutinosa* roots following the steam processing [16, 20]. Two major active compounds in unprocessed *R. glutinosa* roots are iridoid glycoside and phenylethanoid glycoside, which can be degraded due to high temperature to producother constituents. For example, steaming process converted catalpol (iridoid glycoside) into 1,5-dialdehyde to also form other polymers [21). Verbascoside (phenylethanoid glycoside) could be isomerized by high temperatures into iso-verbascoside to make cistanoside F, hydroxytyrosol, and verbascoside by hydrolysis reaction [21]. According to these facts, both catalpol and verbascoside most probably will not be detected in processed *R. glutinosa* [20]. In addition, steam processing also triggers the conversion of glucose or fructose into 5-Hydroxymethyl-2-furaldehyde (5-HMF) by Maillard reaction [22]. Particularly, in processed *R. glutinosa*, the formation of 5-HMF is mainly through fructose mechanism, which isomerized glucose into fructose, followed by the formation of fructose dehydrate to make 5-HMF via a fructose intermediate [21]. This conversion was confirmed in our study, wherein unprocessed *R. glutinosa* roots showed a negligible amount of 5-HMF, while processed *R. glutinosa* roots showed a higher content of 5-HMF. Surprisingly, between the two processed *R. glutinosa* roots, in vitro-derived*R. glutinosa* roots (PKR) showed a higher 5-HMF content (0.15%) than commercial one (0.05%). According to Chinese pharmacopoeia 2015, processed *R. glutinosa* roots must contain more than 0.1% of 5-HMF, which is in line with the content of PKR samples. This result indicates a good quality of raw *R. glutinosa* roots developed in vitro. To confirm this hypothesis, we compared the quantity of active compounds represented by verbascoside in unprocessed *R. glutinosa* roots in those produced in vitro and the commercially produced ones. The results showed that roots derived in vitro have higher verbascoside content (1.725 mg/g) than commercially produced roots (1.22 mg/g).

In this study, we carried out an antioxidant test using the widely known DPPH method. The DPPH antioxidant assay helps in evaluating the capacity of a test sample to scavenge free radicals [13]. The DPPH antioxidant test on all *R. glutinosa* root samples indicated the presence of antioxidant properties. Surprisingly, the processed *R. glutinosa* root samples (PCR and PKR) showed higher antioxidant activity than the unprocessed sample (UPR). This result indicates the positive effect of steam processing on the antioxidant properties of *R. glutinosa* roots. A previous study also reported that there is a positive correlation between steam-drying time and the antioxidant activity of processed *R. glutinosa* roots [23]. The increase in antioxidant activity of processed *R. glutinosa* roots is in accordance with their higher 5-HMF content than the unprocessed *R. glutinosa roots*. Previous studies have reported that the compound 5-HMF and its derivatives exhibit a good amount of antioxidant activity tested by different methods, such as DPPH, ferric-reducing antioxidant power (FRAP), oxygen radical absorbing capacity (ORAC), and computational approaches [24–26]. In addition, the antioxidant capacity of in vitro-derived processed *R. glutinosa* roots (PKR) was higher, though not significantly different from the commercially produced ones (PCR). This could also be correlated with the 5-HMF content that is greater in PKR than in PCR samples. However, compared to unprocessed *R. glutinosa* roots, the study of antioxidant activity of processed *R. glutinosa* roots is still very limited and requires a more comprehensive evaluation.

Iridoid glycosides (e.g., catalpol and harpagide) are one of the predominant compounds in fresh *R. glutinosa* roots, but this group of compounds exhibits a relatively low antioxidant activity [27, 28]. On the other hand, the presence of antioxidant properties in unprocessed *R. glutinosa* roots could mainly be attributed to other bioactive compounds, such as phenolics and flavonoids, both of which are perceived as plant secondary metabolites beneficial to health for their antioxidant and antimicrobial properties [9, 29, 30]. Based on this hypothesis, we evaluated the total phenolic and flavonoid compounds in all *R. glutinosa* samples. Interestingly, our results showed a linear correlation between the total phenolic and flavonoid levels and the antioxidant activities of all *R. glutinosa* extracts. The total phenolic and flavonoid content increased significantly from UPR to PCR and then in PKR samples. This high correlation may confirm that in addition to 5-HMF, phenolic and flavonoid compounds are also responsible for the antioxidant properties of all *R. glutinosa* root samples. Previous studies on the antioxidant activity and total phenolic and flavonoid content in fresh *R. glutinosa* roots showed similar results [30]. In addition, steam processing increased the total phenolic and flavonoid content in *R. glutinosa* roots, as shown by its higher content in processed *R. glutinosa* samples (PCR and PKR) compared to unprocessed roots (UPR). Moreover, between two processed *R. glutinosa* roots, in vitro-derived*R. glutinosa* roots (PKR) showed a higher total phenolic and flavonoid content than the commercially produced samples (PCR). This shows that the *R. glutinosa* roots developed in vitro produce more phenolic and flavonoid compounds indicating a good quality of raw material for medicinal use.

The fresh roots of *R. glutinosa* are known for their anti-inflammatory activity in traditional medicine [16, 31, 32]. However, to date, there have been no comprehensive studies on the anti-inflammatory activities of processed *R. glutinosa* roots. NO exhibits a notable function in biological defense [33], but surplus levels of NO can trigger inflammatory development, such as autoimmune disorders, arthritis, and rheumatoid [34]. Hence, avoiding excessive NO production is one of the main objectives of anti-inflammatory treatment. Therefore, we examined whether processed *R. glutinosa* reduced NO accumulation and iNOS protein expression in LPS-triggered inflammation. Processed *R. glutinosa* samples (PCR and PKR) were found to be more effective than unprocessed *R. glutinosa* roots in inhibiting NO production (Figure 7). These results strengthened other parameters (5-HMF content, antioxidant activity, total phenolic content, and total flavonoid content); as a result, processed *R. glutinosa* roots possess better chemical properties and bioactivity than unprocessed *R. glutinosa* roots. Therefore, we continued our study for the anti-inflammatory evaluation using only processed *R. glutinosa* samples. Western blot analysis showed that iNOS expression gets greatly suppressed in both processed *R. glutinosa* root samples (PKR and PCR). This indicates that processed *R. glutinosa* roots can reduce NO production by downregulating the iNOS expression. However, both PKR and PCR samples showed similar levels of iNOS and NO inhibition, indicating a similar anti-inflammatory effect.

**Figure 10:**
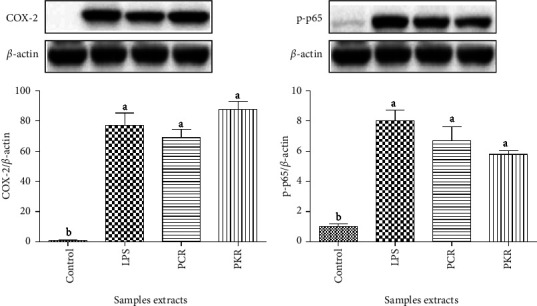
The effect of *R. glutinosa* sample extracts on LPS-induced COX-2 expression and NF-kB activation. (a) Effect of *R. glutinosa* sample extracts on LPS-induced COX-2 expression, (b) effect of *R. glutinosa* sample extracts on LPS-induced and NF-kB activation via p65 phosphorylation. PCR, processed commercial *R. glutinosa*; PKR, processed in vitro regenerated *R. glutinosa*. Values are presented as means ± standard deviation. Same letters are not significantly different by Tukey's test and *p*=0.05. Control images were re-used for illustrative purposes.

In addition, NF-*κ*B and proinflammatory cytokine COX-2 are other key players in the transcriptional processes involved in the inflammation activity. However, both processed *R. glutinosa* samples (PKR and PCR) showed no inhibition activity on COX-2 expression and NF-*κ*B activation via the p65 phosphorylation pathway (Figure 10). The activation of NF-*κ*B leads to the expression of proinflammatory mediators and cytokines, such as NO, COX-2, and iNOS [33]. NF-*κ*B belongs to a family of dimeric molecules with proinflammatory and anti-inflammatory effects. In mammals, the NF-*κ*B group includes five proteins, namely, Rel A (p65), Rel B, c-Rel, NF-*κ*B1 (p50/105), and NF-*κ*B2 (p52/100) [35, 36]. Out of these five, the most active proinflammatory complex is p65/p50 heterodimer. The p65 DNA binding and nuclear translocation commonly lead to NF-*κ*B pathway activation [37]. Though PCR and PKR samples showed no inhibition effect on p65 expression, we suspect that both PCR and PKR could suppress iNOS expression via other NF-*κ*B signaling pathways, such as p38 mitogen-activated protein kinase (p38 MAPK), c-JunN-terminal kinase (JNK) [38], and Syk-mediated activation of PI3K-IKK-I*κ*B signaling pathways [39]. Nevertheless, processed *R. glutinosa* samples (PCR and PKR) possess good anti-inflammatory activity (with similar effect) via the inhibition of LPS-induced iNOS expression.

## 5. Conclusion

In this study, we investigated and compared the 5-Hydroxymethyl-2-furaldehyde (5-HMF) content, total flavonoid and phenolic content, antioxidant activity, nitric oxide (NO) levels, and anti-inflammatory activity of in vitro regenerated *R. glutinosa* roots (PKR) and commercially produced *R. glutinosa* samples (PCR). Compared to unprocessed *R. glutinosa* roots (UPR) and PCR, PKR samples showed a higher content of 5-Hydroxymethyl-2-furaldehyde (5-HMF), flavonoids, and phenolics. Meanwhile, for antioxidant activity and NO levels, PKR and PCR samples showed similar effects, although both were significantly better than UPR. In addition, PKR and PCR samples also showed good anti-inflammatory activity, as they exhibited a similar ability to suppress the expression of iNOS. Therefore, PKR is in line with the Chinese pharmacopeia standards in terms of its 5-HMF content and biological activities. This study is expected to help verify the efficacy of oriental medicines derived from plant materials [40].

## Data Availability

All data used to support the findings of this study are available from the corresponding author upon request.
